# Automated Open-Hardware Multiwell Imaging Station for Microorganisms Observation

**DOI:** 10.3390/mi13060833

**Published:** 2022-05-26

**Authors:** Alain Gervasi, Pierre Cardol, Patrick E. Meyer

**Affiliations:** 1 Genetics and Physiology of Microalgae, InBios/Phytosystems, Institut de Botanique, University of Liège, 4000 Liege, Belgium; gervasialain@hotmail.be; 2 Bioinformatics and Systems Biology Lab, InBios/Phytosystems, Institut de Botanique, University of Liège, 4000 Liege, Belgium

**Keywords:** microscopy, bio-automation, open-hardware, edge computing, IoT, microbiology

## Abstract

Bright field microscopes are particularly useful tools for biologists for cell and tissue observation, phenotyping, cell counting, and so on. Direct cell observation provides a wealth of information on cells’ nature and physiological condition. Microscopic analyses are, however, time-consuming and usually not easy to parallelize. We describe the fabrication of a stand-alone microscope able to automatically collect samples with 3D printed pumps, and capture images at up to 50× optical magnification with a digital camera at a good throughput (up to 24 different samples can be collected and scanned in less than 10 min). Furthermore, the proposed device can store and analyze pictures using computer vision algorithms running on a low power integrated single board computer. Our device can perform a large set of tasks, with minimal human intervention, that no single commercially available machine can perform. The proposed open-hardware device has a modular design and can be freely reproduced at a very competitive price with the use of widely documented and user-friendly components such as Arduino, Raspberry pi, and 3D printers.

## 1. Introduction

As biological researchers often work with microscopical cells, a magnification tool is essential when a direct observation of the organisms is required. The bright field microscope is the most commonly used microscope. It has many applications in a biology laboratory: phenotyping, cell counting, reproduction cycle study, mutants screening, culture monitoring, etc. This widespread technology has existed since the 17th century [1] and has since been improved in new modern forms, increasing quality and magnification [2]. The use of more efficient optical solutions and different light sources or filters allows creating different microscope variations, such as phase contrast, polarization, fluorescence, confocal, and many more [3].

Sample preparation and micrograph analysis is usually time-consuming, which prevents large throughput analysis. The emergence of computerized solutions allowed to greatly improve scalability issues. Sample preparation can nowadays be automated by robots, while computer vision algorithms can analyze micrographs [4,5]. State of the art professional microscopes can offer impressive characteristics [6], but their price might be out of reach of laboratories with modest revenues. Such devices also mainly rely on proprietary “closed source” technologies, thereby preventing the user from modifying and improving his experimental pipeline. The maker and Do It Yourself (DIY) movement allows mitigating those accessibility and adaptability issues [7]. Researchers now have access to open-source solutions whose source files are publicly available, while being able to freely modify the machines to fit their needs [8]. Such systems mainly rely on cheap and broadly available electronic components such as Arduino [9] or Raspberry Pi [10], and on manufacturing technologies like 3D printing [11]. As a result, numerous open-source microscopy systems recently emerged.

In order to study microalgae morphology and motility, we aim to build a machine that should be able to study free moving cells in liquid medium while being able to control the light quality and intensity. The machine should be able to automatically analyze and quantify the motility using computer vision, as this task is complex and time-consuming for a human to perform. Sample parallelization is also required either to study the effect of gradients (light intensity or concentration of a chemical compound) or to work with different strains/species at once. The robot also needs to have liquid handling capabilities, either for sample preparation or reagent addition. Finally, the system should be modular and easily modifiable. For example, various sensors or light sources should be easily added in the future. Such requirements did not meet any commercial solution specifications, a DIY design is, thus, the only solution.

There is a large variety of open-source DIY microscopy systems. Some projects offer inexpensive alternatives to traditional microscopy [12,13,14], systems that put emphasis on portability [15] or modularity [16], allow performing fluorescence microscopy [17,18,19,20], or even use computer vision with machine learning algorithms [21,22,23]. Although the slide/coverslip setup allows for producing high quality images and is also perfectly adapted for a small number of samples, this approach tends to be limited when the experiment requires samples parallelization or automated samples preparation. In addition, cells are only able to move in two dimensions, which could affect their swimming speed and prevent the observation of the normal cell behavior. On the other side, microfluidic apparatus could solve the sampling issue while allowing automatic production of micrographs [24]. This technique is adapted to extract morphological characteristics of the microalgae at high throughput, but the fabrication of such a device requires expensive equipment. Studying different species, strains, or culture conditions in a single run as well as performing motility analysis is, however, impossible using this approach. The parallelization issue can be solved by placing the samples in multiwell plates while having a microscope mobile relative to the samples. We found some interesting robots in the literature [25,26] able to carry out microplate reading. Unfortunately, their design does not allow us to easily adapt the machine to work with different types of multiwell plate or to add additional sensors. The Table 1 summarizes all the desired characteristics that led to the conception of the new device presented in this paper.

In this paper, we describe the conception of a low-cost open-source and modular autonomous multiwell analysis station. Rather than setting up a cumbersome and expensive workflow of specialized tools, we made a multipurpose liquid handling and cell imaging device powerful enough to perform the desired task while being a magnitude cheaper than its commercial counterparts. This machine has been initially designed and tested on microalgal cells to study their morphology and motility. However, it has also been designed with modularity and adaptability in mind, in order to allow applications focused on other organisms and/or different experiments.

## 2. Materials and Methods

Our device is composed of four parts: the chassis, the camera, the sampling system, and the lighting system. The device also benefits from four automation and control tools to perform its task seamlessly, namely a remote control, an automation scripting interface, an autofocus procedure and an image analysis hardware and software.

### 2.1. Hardware Description

#### 2.1.1. Implementation and Design

**Figure 1:**
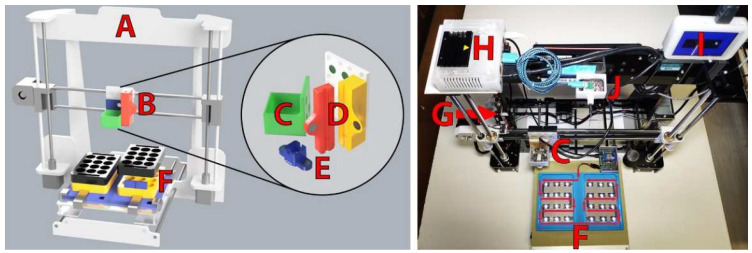
General design of the device. Our machine has been made from the chassis of an AnetA8 3D printer (A) whose plastic extruder has been removed and replaced by a magnetic support (B) to install a digital camera (C) and a lens (E) to obtain an optical magnification of 50×. A support for a micropipette tip has been added (D) to allow deposition of samples in two multi-well plates of twelve wells with transparent bottom (F) fixed on the bed of the 3D printer above 24 RGBW addressable LEDs (SK6812). The motherboard of the printer (G) has been modified to be controlled by a single board computer (Jetson Nano) (H) which captures the image of the camera and displays it in real time on the LCD screen (I). A second security camera (J) has also been added to monitor the remote operations.

Unlike traditional bright field microscopes where the sample is mobile relative to the eyepiece, our design is able to move the camera above the sample (Figure 1). The camera moves along two axes (*X* and *Z* axis) while the sample holder has one degree of freedom (*Y* axis). By having a mobile camera, numerous samples can be analyzed in parallel.

Rather than build a CNC (computer numerical control) machine from scratch, we decided to use the chassis of an AnetA8 FDM (fused deposition modeling) 3D printer [27]. Such a device can be easily controlled by computer and is designed to move a tool (a plastic extruder in the case of a 3D printer) in three dimensions with high precision and repeatability [11]. In addition to being one of the cheapest 3D printer kits on the market, its working surface is relatively wide (220 × 220 × 240 mm) and its electronics are easily accessible and modifiable. Apart from the magnetic tool holder that has been specifically designed to fit on the AnetA8 (Figure 1) to replace the plastic extruder by our microscope and pipette holder, the rest of the system (microscope, pumps, and sample holder) can be installed on virtually any Cartesian 3D printer without requiring complex hardware or software modifications.

All the 3D files, firmware, script, and building and operating instructions are available in the following repository: https://github.com/botabotlab/AMIS (accessed on 5 April 2022).

#### 2.1.2. 3D Printer Modification

**Figure 2:**
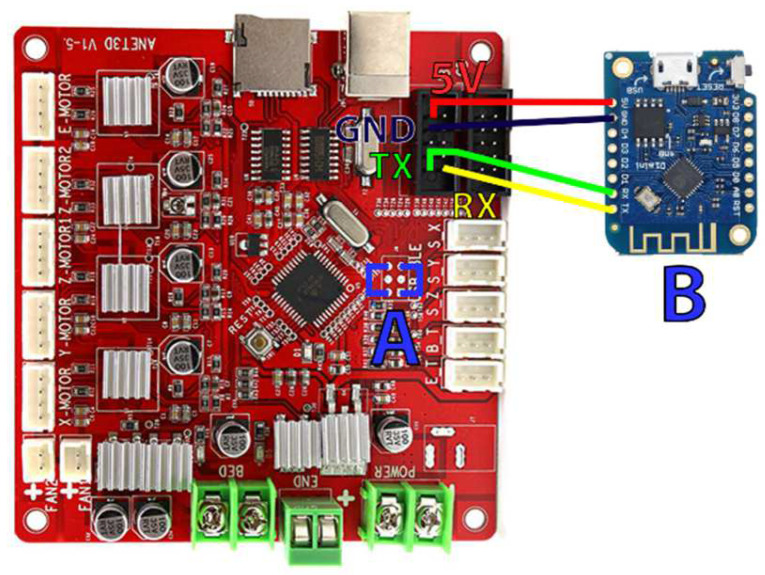
Modification of the Anet A8 motherboard. This modification allows driving the 3D printer in MQTT via its serial port. The motherboard has been set in “Serial” mode by bridging two pads (A) and then an ESP8266 (B) has been connected to the VCC, GND, TX, and RX of the motherboard. It is worth noting that the serial connection with the microcontroller must be “crossed”, meaning the TX of the ESP8266 connects to the RX of the motherboard and the RX connects to the TX.

Remote control of the printer chassis has been made possible by connecting an ESP8266 microcontroller to the serial port of the motherboard (Figure 2). This modification allows a bidirectional communication between the printer and the automation software using the MQTT protocol [28]. The microcontroller is connected to the motherboard using an FC10P connector and can easily be removed to restore the printer’s original functions.

#### 2.1.3. Camera

**Figure 3:**
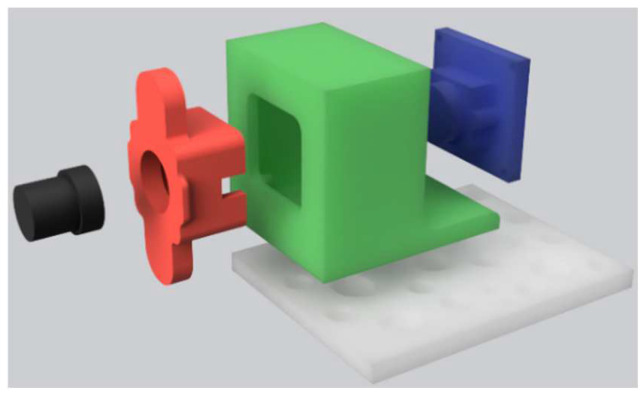
Digital microscope and its magnetic support. The microscope is composed of a photo sensor (blue) fixed on the green support with four screws, as well as the lens support (red) placed in front of the sensor and holding an inverted camera lens (black) that allows a 50× optical magnification. The camera is placed on the white magnetic support fixed on the *X* axis of the 3D printer. The camera support has been 3D printed in translucent ABS using a Prusa I3 MK3 (nozzle: 250 °C, Bed: 100 °C, nozzle: 0.4 mm, layer height: 0.2 mm, filling: 100%). The lens must be perpendicular to the sensor to avoid optical aberrations on the edge of the images.

The plastic extruder of the printer has been removed and replaced by a 3D-printed magnetic fixation that allows the placement of a digital microscope made from a modified webcam module (Figure 3). For the optical magnification, we have used a similar setting than in [29] by placing a reversed camera lens in front of a camera sensor. A 2mpx camera module has been used in combination with different M12x0.5 CCTV camera lenses with focal length ranging from 2.8 mm to 16 mm. All the lenses worked without requiring any adjustment of the adapter and allowed for a magnification of up to 50× (and up to 100× with digital magnification).

#### 2.1.4. Sampling

**Figure 4:**
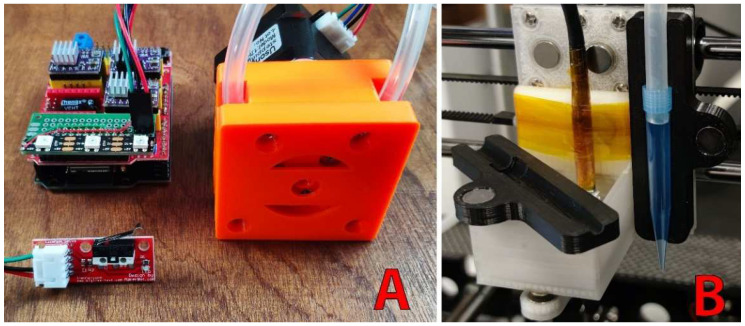
Automatic sampler. The sample preparation system is composed of a 3D-printed peristaltic pump [30] (orange; **A**) connected to a micropipette tip held by a magnetic support (black; **B**) placed next to the camera (white; **B**).

In addition to image capture and analysis, the microscope is able to prepare samples using a DIY sampling system based on a 3D printed peristaltic pump that we previously developed [30]. A micropipette tip (P1000) can be placed next to the camera in a magnetic holder and connected to a silicone tube (Figure 4). The pump input can be connected to an Erlenmeyer flask to quickly fill the plates with a defined volume (which is faster than doing it manually with a micropipette). When the pump is connected to a photobioreactor, samples can be extracted at defined times in order to capture the culture state at different stages of growth or even to follow the impact of a change of culture conditions (light intensity or wavelength, temperature, nutrient concentration, CO_2_ concentration in air, etc.). The peristaltic pump has a relative error of 3.5% for volumes of 1 mL [30] and is suitable for filling wells, and small inaccuracies are compensated by the autofocus.

#### 2.1.5. Sample Holder and Illumination System

**Figure 5:**
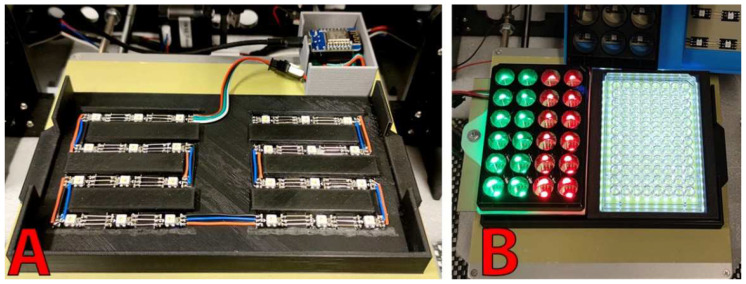
Sample holder and lighting systems. RGBW LEDs were mounted on the sample holder to illuminate individual wells of two 12-well clear-bottom plates (**A**); 24- or 96-well plates can also be used (**B**).

The preparation of a slide/coverslip assembly as well as the treatment of the used slide being difficult to automatize, we decided to work in liquid phase with 1 mL samples that are placed in two multi-well plates with transparent bottoms (Cellvis, P12-1.5H-N) for a total of twenty-four slots (Figure 5A). The sample holder is composed of a 3D printed support (files available in the repository) that hold the multi well plate at defined positions on the *Y* axis of the chassis. This approach allows the treatment of several samples in a row and facilitates the waste treatment. The plates being removable, it is possible to start a new run in a few seconds by replacing the plates, strongly limiting human interventions. Image capture is also facilitated because the microscope is able to move above the sample to perform autofocus and automated image capture.

The samples are illuminated from the bottom of the transparent wells using RGBW (634 nm, 522 nm, and 463 nm) LEDs (SK6812) shown in Figure 5. Installation of these LEDs requires only three wires (+5 V, GND, and Data) and can be soldered in series to reach hundreds of LEDs. Intensity (255 levels) of each of the four channels (red, green, blue, and cold white) of each LED can be individually adjusted. This property can be used to illuminate the samples with a certain color to improve the contrast, to test the effect of light intensity or light color on microalgae, or to turn on the light at the time of image capture to avoid phototropic or photophobic response that is susceptible to disturb the measurement.

Since the multi-well plates have standardized dimensions and regular spacing between the wells, adaptation of the machine to work with 24- or 96-well plates is possible (Figure 5B). The user can choose the type of plate to use, and the camera and micropipette tip positions will be automatically recalculated by the robot. A manual offset can be added to compensate in case of LED or support misplacement. The position of the wells can also be manually entered (in the form of a JSON file) if a custom support is used. The 96-well plate support currently uses a white LED panel instead of individually addressable RGBW LED.

### 2.2. Automation and Control

#### 2.2.1. Remote Control

MQTT (Message Queuing Telemetry Transport) [28] is a publish/subscribe machine to machine communication protocol invented by IBM. Initially designed to retrieve information from remote locations from resource constrained devices (such as a microcontroller) over an unreliable, high latency, and low bandwidth connection (satellite connection for instance) this protocol is nowadays mainly used for IoT (Internet of Things) applications. This widely used protocol offers numerous advantages for our application. It is simple to use, secure (TLS encryption), reliable, lightweight (in terms of computing resources and bandwidth usage), and highly scalable. This protocol is furthermore compatible with a large variety of programming languages (python, C, C#, C++, java, javascript, lua, ruby, rust, etc.).

**Figure 6:**
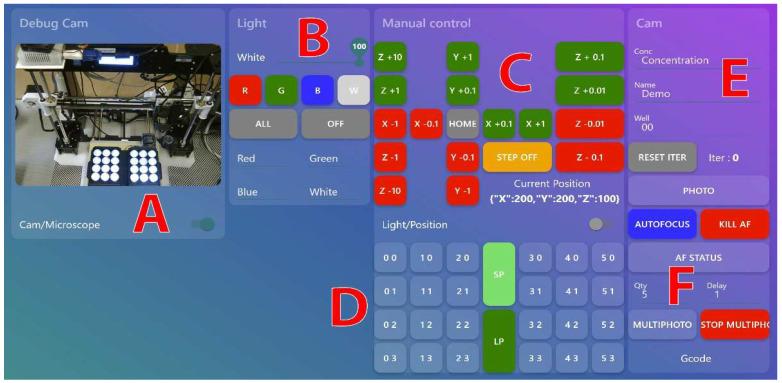
Node-RED dashboard. The graphical user interface is composed of four panels (debug cam, light, manual control, and cam). The first panel (**A**) allows access to the security camera (Cam) to remotely view the machine to ensure its proper working or to access the microscope image (Microscope). The second panel (**B**) allows controlling the light. The control panel (**C**) allows manually moving the camera on its three axes and returns the current position of the camera. The plate menu (**D**) allows moving the camera to various pre-programmed positions, and turning on the light in individual wells. The “SP” (safe position) button sends automatically the camera to the upper right corner of the robot and advances the plate towards the user, thus facilitating the installation of multi-well plates, while the “LP” (last position) button returns the camera to its previous position (in case the user accidentally moves the camera). The panel (**E**) allows capturing and naming of images. Finally, panel (**F**) performs the autofocus.

The microscope control system is using the open-source home automation software Node-RED [31]. Node-RED is user-friendly, and it facilitates usage and automation of the device. This low code software built on NodeJs supports the MQTT protocol and can be used to easily handle the automation thanks to its flow programming paradigm. Furthermore, it allows a straightforward development of web-based GUI accessible via a smartphone or a PC (Figure 6). In other words, users can manually and remotely control the robot from the web interface to collect samples, control the light or take pictures.

#### 2.2.2. Automation

To facilitate the automation for the users unfamiliar with Node-RED, we developed a scripting system based on a JSON structure that is both intuitive and flexible (Table 2). The user can, thus, define the sequence name, the need to purge the circuit, the wells to fill and/or capture if desired, the light parameters, and the time between two samplings or captures. Once entered in the interface, the validity of the sequence will be checked. An error will be returned indicating the missing property in case of an erroneous or incomplete script. If valid, the program will return a required time estimation. Once the sequence is finished, the robot will turn off the lights, place the machine in a safe position, and return the elapsed time (it is also possible to add a push or email notification to inform the user that the experiment is finished).

#### 2.2.3. Autofocus

**Figure 7:**
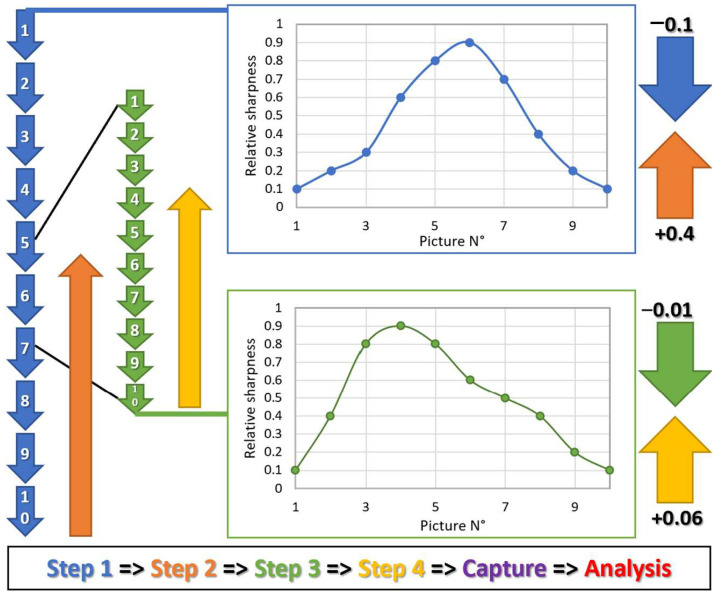
Autofocus. The autofocus procedure consists in measuring the sharpness of 20 images captured at different heights to determine the optimal camera positioning. The arrows schematize the vertical movement of the camera (in mm) while the plot represents the “relative sharpness” of the capture returned by the OpenCV Laplacian function.

As the sample volume is known by the robot, placement of the camera at a theoretical optimal focusing position could theoretically be possible but since the volume of the sample can slightly vary due to inaccuracies in the plate positioning, wells filling, or because algae can concentrate at variable heights in the wells, an autofocus step is nevertheless necessary. This task is performed using an OpenCV [32] script that takes advantage of the Laplacian function [33] capable of measuring the sharpness of an image. The autofocus procedure takes place in two phases (Figure 7). First, the camera is moved 0.5 mm above the theoretical “optimal” focus height that depends on the known volume of the sample. The camera is then moved down ten times by 0.1 mm increments. A sharpness evaluation is performed at each level. The script determines the height with the highest score and moves the camera 0.05 mm above this height. These steps are then repeated using 0.01 mm increments. Finally, the camera moves to the position that produces the highest sharpness value.

As the liquid can move freely in the wells, the observation of the cells could be disrupted by liquid agitation. It is worth noting that there are typically multiple focus points because the liquid column is large (several mm) and the algae are often distributed throughout the liquid. To overcome these inconveniences, we have made sure that the microscope only captures the upper layer of liquid (generally where the image is the sharper), and to introduce a slight delay between the movement of the *Y* axis and the capture of the picture to allow for stabilization of the medium. Other systems could have been considered, but this approach has proven to be functional and is the most effective in terms of simplicity and working speed.

#### 2.2.4. Image Analysis

The device chosen to perform the microscope automation and images analysis is an Nvida Jetson nano development board [34] running on the Linux operating system (Ubuntu). This powerful board integrates 128 Cuda cores in addition to a quite efficient 64-bit CPU allowing it to integrate more complex image recognition algorithms in the future and even to implement deep learning applications. We tested other single board computers, such as the well-known Raspberry Pi 3 [10], but the speed/frame rate of the image analysis was not as good. Python 3 and the open-source computer vision library OpenCV [32] were used to perform the autofocus as well as the images/video analysis.

**Figure 8:**
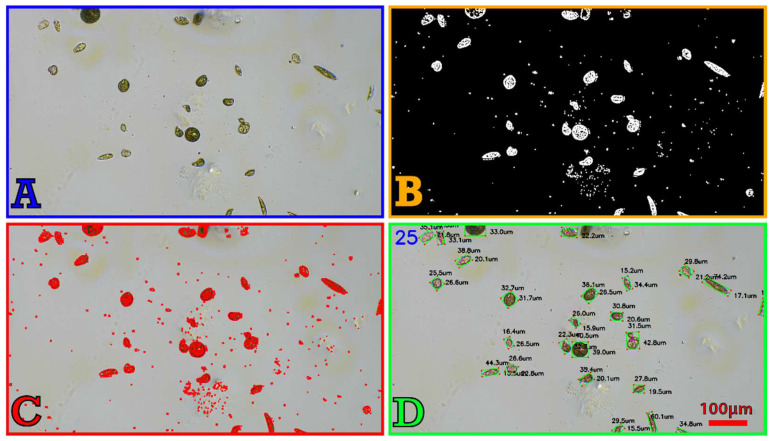
Image analysis process of an *E. gracilis* sample. An image is captured after performing the autofocus (**A**) and is converted to a binary thresholded image (**B**) used for the edge detection (**C**). The areas lower than a predefined threshold are removed and the annotation (number of cells and dimensions) are added to the image (**D**).

The image analysis (Figure 8) allows the counting of cells and extraction of phenotypic features of those cells (size, shape, and color). It is accomplished in four steps. Once an image is captured, that is after the autofocus, it is converted to grayscale. A thresholding [35] step is then applied and consists in converting the grayscale image (the pixels can take any value between 0 and 255) into a binary image (the pixels take the value of 0 or 255). All pixels below the threshold become completely white, and those above become black. An erosion [36] pass is then applied to enlarge the areas with white pixels to “connect” nearby pixels and “close” the cells. We then use the edge detection function [37] of OpenCV. Although the previous steps have greatly cleaned up the image, some particles and artifacts might persist and can sometimes be detected as edges. These imperfections typically have a small surface area and can easily be ignored by the last step that removes all the area lower than a defined threshold. Annotations (number of cells, width and height of each cell) are then added to the final image. The script can work in real time on a live video and can return all the measurements (e.g., export the length and width of each cell in a csv file).

To test our microscope, we used two model organisms widely used in the laboratory: the unicellular and flagellated green alga *Chlamydomonas reinhardtii* [38] that has a diameter generally between 5 µm and 12 µm, and the flagellated excavate *Euglena gracilis* that has a typical length of around 100 µm [39]. Both algae were grown with a constant light of 100 µE m^−2^ s^−1^ and at a temperature of 22 °C in liquid TAP medium in presence of vitamins (biotin 10–7%, B12 vitamin 10–7% and B1 vitamin 2 × 10^−5^% (*w*/*v*)) [40]. Different substances known to have an effect on microalgae growth [41,42,43,44] have been added to the cultures in order to test the ability of our microscope to observe morphological alterations of the cells.

## 3. Results

### 3.1. Hardware and Software Validation

#### 3.1.1. Magnification

**Figure 9:**
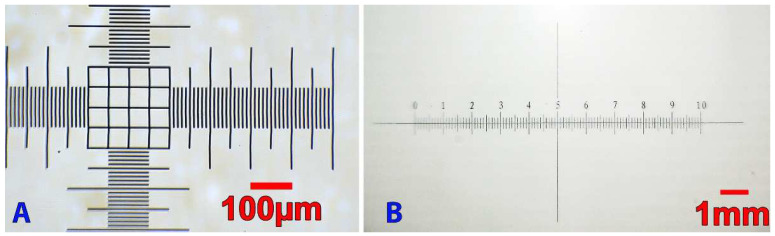
Microscope calibration of two lenses with a 1920 × 1080p camera. The 2.8 mm lens (**A**) was calibrated with a 10 µm/division slide and the 16 mm lens (**B**) has been calibrated with a 100 µm/division reference slide.

A calibration is mandatory if the user wants to measure the size of a cell and should be performed when the resolution of the camera is modified or when the lens is changed. Since the lenses have a fixed focal length, it is possible to calibrate several lenses beforehand and import a predefined calibration setting. Calibration consists of converting a distance in pixels into a distance in micrometers and can, therefore, be performed with any image processing or analysis software (imageJ, Adobe photoshop, and even Microsoft paint) or with an OpenCv python script allowing the measurement of a distance between two points. The calibration (Figure 9) of the 2.8 mm focal length lens with a 10 µm/division calibration slides allows us to obtain the calibration value of 0.5 µm/pixels with a 1920 × 1080p image (and 0.75 µm/pixels for 720p), while the calibration of a 16 mm lens (with a larger field of view at the cost of a lower magnification) with the 100 µm/division produced a calibration value of 7.46 µm/pixels (11.16 µm/pixels for 720p).

**Figure 10:**
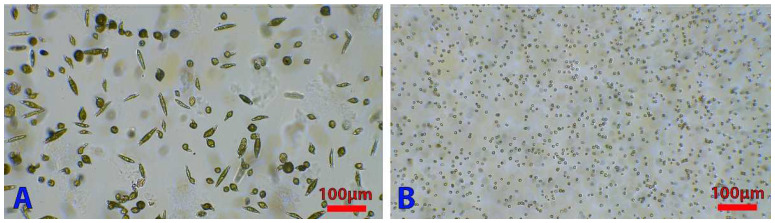
Examples of micrographs. *Euglena gracilis* (**A**) and *Chlamydomonas reinhardtii* (**B**) micrographs captured by our DIY microscope using a 2.8 mm lens that produced a 50× magnification.

The distance between the sensor and the lens is not very important, since it is possible to have a focused image by moving the camera relative to the samples. The quality of the magnification is sufficient to perform a cell count or to dissociate cell shapes (Figure 10) and equals 50× magnification of commercial bright field microscopes. Our system has the advantage of being cheap and easy to implement.

#### 3.1.2. Positioning Repeatability

**Figure 11:**
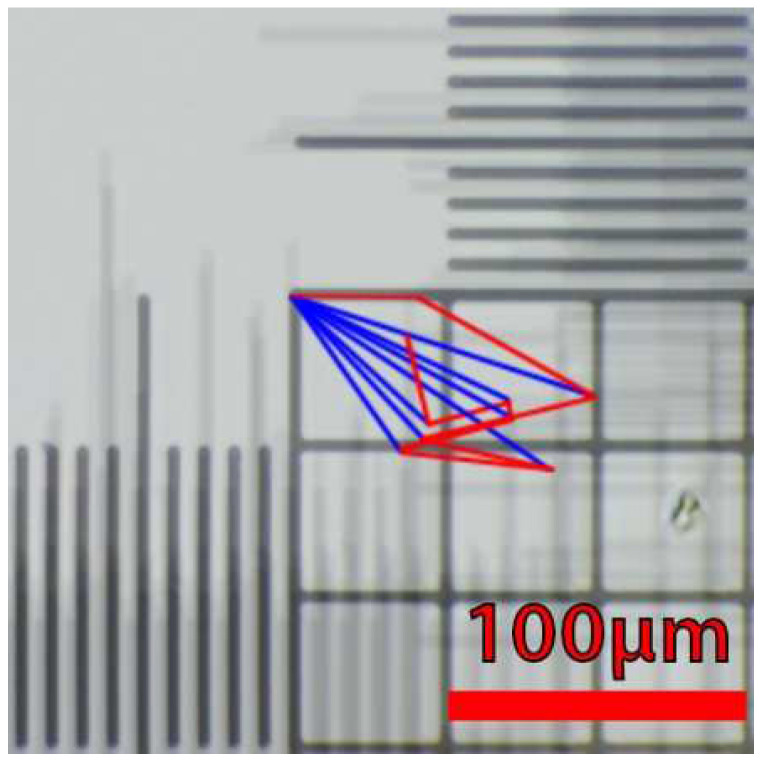
Evaluation of the repeatability of the camera positioning. Ten images have been captured and superimposed after homing and successive repositioning of the camera. The blue lines represent the deviation from the initial position and the red lines represent the deviation from the previous position.

**Figure 12:**
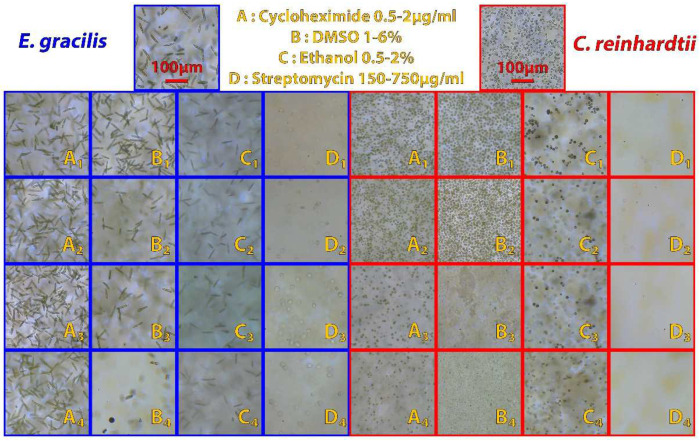
Observation of the evolution of morphology of algal cells exposed to chemicals. Briefly, 2 mL cultures of *E. gracilis* (blue) and *C. reinhardtii* (red) with an initial concentration of 10^5^ cells/mL were exposed during 10 days to different concentrations of cycloheximide (0.5 µg/mL, A1; 1 µg/mL, A2; 1.5 µg/mL, A3; 2 µg/mL, A4), DMSO (1%, B1; 2%, B2; 4%, B3; 6%, B4), ethanol (0.5%, C1; 1%, C2; 1.5%, C3; 2%, C4), and streptomycin (150 µg/mL, D1; 350 µg/mL, D2; 500 µg/mL, D3; 750 µg/mL, D4). Top pictures: control cultures. In brief, most chemical concentrations slow growth of both algae; DMSO modifies the length of *E. gracilis* cells; presence of ethanol causes bleaching of *E. gracilis* cells and streptomycin causes the appearance of colorless spheroids in *E. gracilis*.

The camera’s positioning repeatability was tested by placing a calibration slide with graduations separated by a defined space (10 µm/division) under the microscope (Figure 11). An image was captured after the focus was adjusted. The camera was sent to the *X*0, *Y*0, *Z*0 position (home) and then returned to the previous position (to move the 3 axes in both directions to account for any backlash) and a second image was captured. This procedure was executed 10 times. The images were then superimposed to evaluate the 3-axis deviation. The average camera positioning’s deviation after homing for the *X* axis is 37 µm (±22 µm), 14 µm (±11 µm) for the *Y* axis, and 9 µm (±2 µm) for the *Z* axis (Figure 12). The maximum deviation from the origin for the *X* axis is 63 µm, 32 µm for the *Y* axis, and 11 µm for the *Z* axis. The *X* and *Y* deviations are totally negligible because the studied cells are mobile anyway and the position’s repeatability of the box (±0.5 mm) in its 3D printed holder is much higher than the camera’s one. In the *Z* axis cases, the deviation of ±9 µm is sufficient to lose the image’s focus, but as it remains within the autofocus algorithm displacement range of 1 mm, the small inaccuracies in positioning will, thus, be easily compensated.

#### 3.1.3. Automation and Remote Control

Regarding the automation, we have tested different scripts that allow covering our typical use cases: taking pictures of pre-filled plates, filling plates, and controlling the light, as well as “complete” automation sequences that combine all of these features with precise timings. The test script in Table 3 has been designed to explore and validate all device functions while using distinct parameters of light color, volume, and sample position. Once started from a smartphone connected on the cellular network (to confirm the robot ability to be controlled from outside the laboratory local network), we verified that the robot correctly followed the action described in the script and produced the expected output.

The system has correctly followed the script and took 3.6 min to complete (the script has estimated a run time of 3.3 min). The light color, well positions, and delays between samples were respected. The volume of the samples remained in the expected limit of ±3.5% and three pictures adequately named and focused were produced.

Another script (Table 4) has been tested to measure the throughput of the device by filling all the wells of two 12-well plates with 1 mL of samples and taking a picture of each well. We measured the effective time of the procedure and compared it to the theoretical run time of 9.6 min returned by our software.

The system has correctly followed the script and produced 24 pictures in 9.83 min. Slight variations between the expected and observed run time are due to varying time required for the sampling (motor activation) and autofocus (correct camera placement). The robot is still able to provide a realistic time estimation

### 3.2. Biological Validation

#### 3.2.1. Study of Morphological Alteration in Presence of Chemical Compounds

To illustrate the use of the imaging station for large throughput analysis, we carried out experiments to inhibit the growth of microalgae in 12-well plates in the presence of different concentrations of chemical inhibitors (Figure 12) that have been manually added. The observations have been made automatically (autofocus and image capture) after placing the multiwell plates containing the microalgae cultures subjected to the gradient of inhibitors on the 3D printed plate holder and starting the analysis script. In total, eight 12-well plates were used for the experiment (three technical replicates by condition in two biological replicates).

This proof of concept highlights the ability of the microscope at capturing many images automatically and quickly. This information can be used to monitor a culture, detect contamination, measure the growth of an organism, or to set up a machine learning model capable of recognizing a particular condition and automatically notifying the user if necessary. Chemical addition has been performed manually as the system is currently only able to manipulate one type of liquid. For this experiment, the time benefit of an automated chemical addiction would have been marginal compared to the image capture that is the most time-consuming aspect. However, automatic sampling could be used if the experiment requires to handle one type of fluid.

#### 3.2.2. Study of Light Color Dependency Aggregation State of Algal Cells

**Figure 13:**
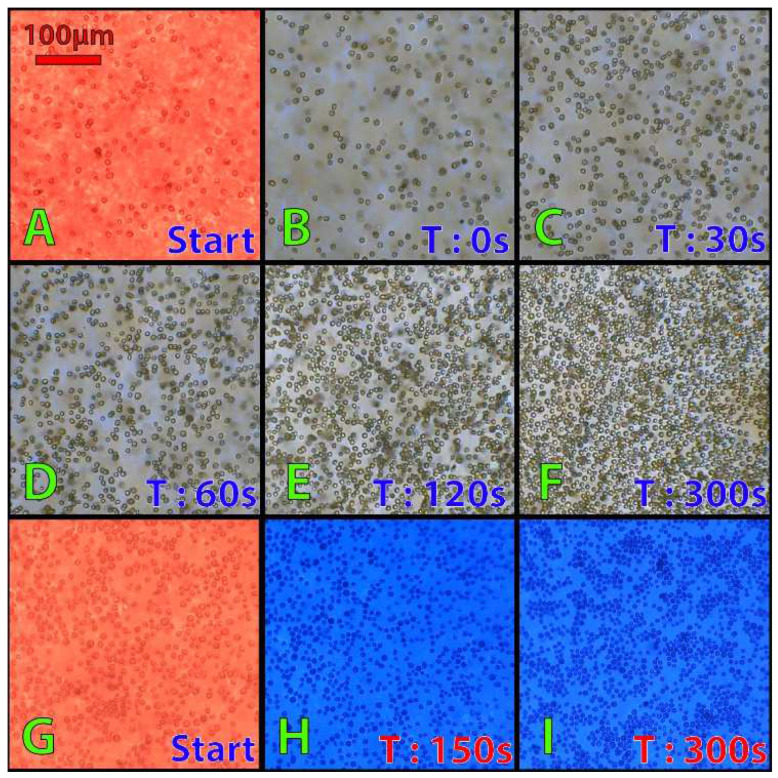
Effect of light color on microalgae motility. Aggregation of *C. reinhardtii* cells (7 × 10^6^ cells mL^−1^) exposed to white light (**A**–**F**) or blue light (**G**–**I**) after a 5 min treatment with red light. The concentration of the culture does not change between the beginning and the end of the experiment, the lower number of cells seen at the beginning of the experiment comes from the fact that the algae are able to swim freely in three dimensions and can, therefore, move away from the camera’s focus.

A second set of experiments were conducted to monitor the aggregation of Chlamydomonas cells as a function of the color of light (Figure 13). As demonstrated by Titus et al. [45], *C. reinhardtii* can reversibly modify the adhesiveness of its flagella depending on the wavelength to which it is exposed. As our microscope is equipped with RGB LED and can observe and record the movement of the algae, our machine was able to reproduce their observations (Figure 13). We observed that if Chlamydomonas cells exposed to red light move freely and quickly and do not interact with each other (no formation of cellular aggregates), the speed of movement of the cells slows down, and the algae start to form aggregates to finally stop moving after 300 s of exposure to white light (Figure 13A–F). This effect can be reproduced using specifically blue light instead of white light (Figure 13G–I).

The experiments confirm the capacity of our microscope to modify and analyze, in real time, the behavior of a microalgae culture. As the machine is equipped with a microcomputer capable of performing image analyses, the microscope would also be able to quantify the changes that take place in the culture. By counting the number of apparent cells as a function of time, or by quantifying the direction and speed of movement of microalgae.

#### 3.2.3. Analysis of Algal Cell Velocity and Trajectory

**Figure 14:**
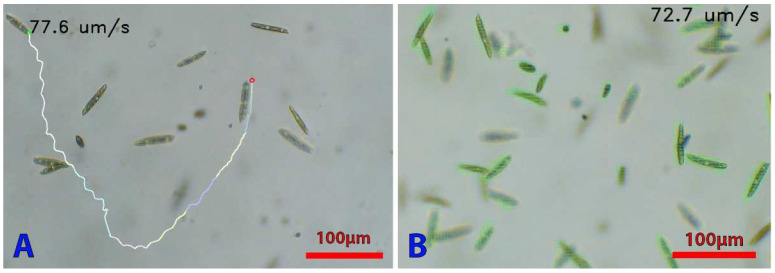
Microalgae tracking. (**A**) Tracking a *E. gracilis* cell to determine its trajectory. Cell concentration in the well was 2 × 10^5^ cells mL^−1^. The tracking is performed with a OpenCV script allowing the user to click on the cell to track. The cell’s path will then be drawn in real time on the live video. (**B**) Analysis of the direction of movement of all the cells in a culture. Green lines allow determining the direction and speed of the algae (the length of the lines is relative to the speed). Both scripts allow obtaining the speed in µm/s.

Motility (velocity and trajectory) is a key parameter to study phenomenon such as phototaxis or chemotaxis, dissociate living/active cells from dead/inactive cells, or perform a screening for organisms with different motility parameters. Since the algae are free to move in three dimensions in the well and the microscope can film the cultures at 30FPS while performing computer vision, a real-time analysis of the motility of the cells is possible. To demonstrate the ability of our device to analyze cell movements, we used optical flow algorithms [46] to track the movement of an individual cell and quantify its swimming speed, as well as highlighting the overall motion of a whole culture (Figure 14).

## 4. Discussion

In conclusion, we designed and built a modular and remotely controllable open-source microscope using low-cost components such as a webcam, a 3D printer, and a single board computer. The machine is capable of automatically extracting samples, autofocusing, and capturing and analyzing an image or video while controlling the light for up to 24 samples in parallel. The system can be used to quickly analyze either a manually collected set of samples in a multi-well plate or to perform continuous culture monitoring when connected to a photobioreactor. The microscope can achieve an optical magnification of 50× while offering images of sufficient quality to analyze the size, shape, or color of a micro-algae or macroscopic organisms when a smaller focal length lens is used. We have performed various biological experiments to test the ability of our machine to highlight changes in algal cell morphology, or to record cell movements with a minimal remote human supervision.

Various improvements of the machine are possible, such as the addition of a higher quality camera module or lenses to increase the image quality and magnification level or the addition of sensors such as a spectrophotometer or a spectrofluorometer to study various parameters (pigments concentration, photosynthesis, and turbidity) thanks to the modular magnetic tool holder. The current device is focused on microscopic observations in liquid medium; macroscopic observation with the 16 mm lens is, however, also possible. Another 3D printed support could be designed to hold Petri dishes to study colonies on a solid agar medium. Machine learning algorithms could be further integrated to improve the image analysis capabilities and to allow the microscope to dynamically control the culture conditions in view of optimizing a precise phenotypic trait. All these modifications are possible thanks to the open-source approach of our project. Indeed, any laboratory is allowed to build, use, modify, and share the machine without being limited by proprietary or cloud-based components or software.

## Figures and Tables

**Table 1:** Evaluation of the pertinence of the different approaches to meet our specifications.

	Automatic Sampling	Autofocus	Image Analysis	Video Analysis	Parallelization	Easily Adaptable
**Traditional microscope**	No	No	Yes	No	No	No
**Automated microscope**	No	Yes	Yes	Low	Low	No
**Microfluidic**	Yes	Yes	Yes	No	No	No
**Microplate imagine**	Yes	Yes	Yes	Yes	Yes	No
**Our device**	Yes	Yes	Yes	Yes	Yes	Yes

**Table 2:** Example of JSON scripts that allows the filling of a plate, taking pictures of a pre-filled plate, or performing these two tasks at once.

Plate Filling Example	Plate Capture Example	Plate Filling and Capture
{“name”: “Fill plate 1”, “pos”:[ [0,0],[1,0],[2,0], [0,1],[1,1],[2,1], [0,2],[1,2],[2,2], [0,3],[1,3],[2,3] ], “sampling”: true, “volume”: [1]}	{“name”: “Capture plate 1”, “pos”:[ [0,0],[1,0],[2,0], [0,1],[1,1],[2,1], [0,2],[1,2],[2,2], [0,3],[1,3],[2,3] ], “capture”: true, “light”: [[0,0,0,255]]}	{“name”: “DemoExp”, “pos”:[ [0,0],[1,0],[2,0], [0,1],[1,1],[2,1], [0,2],[1,2],[2,2], [0,3],[1,3],[2,3] ], “sampling”: true, “capture”: true, “light”: [[0,0,0,255]], “volume”: [1]}

**Table 3:** Example of automation script that allowed us to collect samples and take pictures without requiring any human interventions.

Element	Structure/Description
**Script**	{“name”: “DemoSeq”, “pos”: [[0,0], [2,3], [3,0]], “light”: [[255,0,0,0], [0,255,0,0], [0,0,255,0]], “delay”: [30,60,0], ”sampling”: true, “volume”: [1,2,3], “trash”: true, “capture”: true}
**Red sample**	*1 mL* of culture is collected in the well *0,0* (top left of the plate 1) after *purging* the circuit. The *light* is set to *red* and the image *DemoSeqX0Y0.png* is captured.
**Green sample**	*30 s* after the previous sample, *2 mL* of culture is collected int the well *2,3* (bottom right of the plate 1) after *purging* the circuit. The *light* is set to *green* and the image *DemoSeqX2Y3.png* is captured.
**Blue sample**	*60 s* after the previous sample, *3 mL* of culture is collected int the well *3,0* (top left of the plate 2) after *purging* the circuit. The *light* is set to *blue* and the image *DemoSeqX3Y0.png* is captured.

**Table 4:** Script used to test the throughput of the robot.

Script
{“name”:“MaxThroughput”,“pos”:[[0,0],[1,0],[2,0],[0,1],[1,1],[2,1],[0,2],[1,2],[2,2], [0,3],[1,3],[2,3],[3,0],[4,0],[5,0],[3,1],[4,1],[5,1],[3,2],[4,2],[5,2],[3,3],[4,3],[5,3]],“light”: [[0,0,0,255]], “delay”: [0], ”sampling”: true, “volume”: [1], “trash”: false, “capture”: true}

## Data Availability

Source file repository; https://github.com/botabotlab/AMIS (accessed on 5 April 2022).
